# Altered cerebellar functional connectivity in chronic subcortical stroke patients

**DOI:** 10.3389/fnhum.2022.1046378

**Published:** 2022-11-11

**Authors:** Wenjun Hong, Yilin Du, Rong Xu, Xin Zhang, Zaixing Liu, Ming Li, Zhixuan Yu, Yuxin Wang, Minmin Wang, Bo Yang, Fenfen Sun, Guangxu Xu

**Affiliations:** ^1^School of Rehabilitation Medicine, Nanjing Medical University, Nanjing, China; ^2^Department of Rehabilitation Medicine, Nanjing Drum Tower Hospital, The Affiliated Hospital of Nanjing University Medical School, Nanjing, China; ^3^Department of Radiology, Nanjing Drum Tower Hospital, The Affiliated Hospital of Nanjing University Medical School, Nanjing, China; ^4^School of Biomedical Engineering and Instrument Science, Zhejiang University, Hangzhou, China; ^5^Binjiang Institute of Zhejiang University, Hangzhou, China; ^6^Center for Brain, Mind, and Education, Shaoxing University, Shaoxing, China; ^7^Department of Rehabilitation Medicine, The First Affiliated Hospital of Nanjing Medical University, Nanjing, China

**Keywords:** stroke, cerebellar anterior lobe, cerebellar posterior lobe, resting-state fMRI, functional connectivity

## Abstract

**Background:**

Previous studies demonstrated that cerebellar subregions are involved in different functions. Especially the cerebellar anterior lobe (CAL) and cerebellar posterior lobe (CPL) have been postulated to primarily account for sensorimotor and cognitive function, respectively. However, the functional connectivity (FC) alterations of CAL and CPL, and their relationships with behavior performance in chronic stroke participants are unclear so far.

**Materials and methods:**

The present study collected resting-state fMRI data from thirty-six subcortical chronic stroke participants and thirty-eight well-matched healthy controls (HCs). We performed the FC analysis with bilateral CAL and CPL as seeds for each participant. Then, we detected the FC difference between the two groups by using a two-sample *t*-test and evaluated the relationship between the FC and scores of motor and cognitive assessments across all post-stroke participants by using partial correlation analysis.

**Results:**

The CAL showed increased FCs in the prefrontal cortex, superior/inferior temporal gyrus, and lingual gyrus, while the CPL showed increased FCs in the inferior parietal lobule, precuneus, and cingulum gyrus in the stroke participants compared with HCs. Moreover, the FC alteration in the right CAL and the right CPL were negatively correlated with executive and memory functions across stroke participants, respectively.

**Conclusion:**

These findings shed light on the different increased FC alteration patterns of CAL and CPL that help understand the neuro-mechanisms underlying behavior performance in chronic stroke survivors.

## Introduction

Increasing studies have documented that the cerebellum is engaged in a wide range of tasks, including sensorimotor control, language, spatial, emotional, and executive functions (Stoodley and Schmahmann, 2009; Stoodley et al., 2012). This multifunctional role may be associated with its complex structure. The cerebellum is comprised of ten lobules, grouped as the anterior lobe (lobules I-V), posterior lobe (lobules VI-IX), and the flocculonodular lobe (lobule X) (Stoodley and Schmahmann, 2009). These subregions in the cerebellum respond to differential brain functions by the intrinsic functional connectivity (FC) with the cerebral cortex (Buckner et al., 2011; Yeo et al., 2011). Previous studies have manifested that, in subregions of the cerebellum, the cerebellum anterior lobe (CAL) and the lobule VIII of the cerebellum posterior lobe (CPL) predominantly contribute to sensorimotor function, whereas lobules VI and VII of the CPL are involved in the cognitive function (Schoch et al., 2006; Schmahmann et al., 2009; Stoodley and Schmahmann, 2009). A previous study reported that performance on executive function was associated with damage to the CAL (Stoodley et al., 2016). Moreover, task-dependent fMRI studies have reported that activation in the CAL is positively correlated with the motor performance of finger-tapping (Loubinoux et al., 2007), whereas the activation in the CPL is associated with visuospatial working memory (Nitschke et al., 2005) and language-related activity (Schmahmann et al., 2019).

Recently, with increasing attention to resting-state fMRI, a task-independent and non-invasive method, studies have revealed abnormal brain activity in CAL and CPL in stroke participants. For CAL, stroke participants showed increased FCs between the ipsilesional anterior inferior cerebellum and superior cerebellum (Wang et al., 2010) and between the right anterior inferior cerebellum and left middle frontal gyrus (MFG) (Yin et al., 2014) compared with healthy controls (HCs). While, for CPL, stroke participants showed increased FCs between the ipsilesional CPL and the contralesional primary motor cortex (Yin et al., 2012), and reduced FCs between the bilateral cerebellar lobule IX and the ipsilesional medial part of the prefrontal cortex, dorsolateral prefrontal cortex, lateral parietal cortex (Li et al., 2013), between the right CPL and left precentral gyrus (PreCG), inferior frontal gyrus (IFG), inferior parietal lobule (IPL), middle temporal gyrus (MTG) (Tang et al., 2016), between the bilateral CPL and left IPL (Fan et al., 2019), and between the right cerebellar lobule VI and left PreCG (Zhang et al., 2022) compared with HCs. These findings demonstrated that the spontaneous brain activities in the cerebellar subregions were disrupted by stroke, and the CAL and CPL experienced different functional reorganization patterns after stroke. However, there is currently a lack of studies that have used bilateral CAL and CPL as seeds to systematically explore the FC between the cerebellum and cerebral cortex in chronic stroke participants, and although previous studies have preliminarily explored the relationship between the cerebellum and sensorimotor/executive function and other cognitive functions, the relationship between the FC alterations between the cerebellar subregions and the cerebral cortex and the behavior performances still needs to be further explored.

The present study aims to examine the differences in FC of bilateral CAL and CPL between stroke participants and HCs. Based on the previous findings regarding the abnormal functional activity of CAL and CPL (Wang et al., 2010; Yin et al., 2012, 2014; Li et al., 2013; Fan et al., 2019; Zhang et al., 2022) and their relationships with behavior performances in stroke participants (Nitschke et al., 2005; Loubinoux et al., 2007; Stoodley et al., 2016; Schmahmann et al., 2019), we hypothesized that: (1) the FC of CAL and CPL in stroke participants would show different functional alterations patterns compared to HCs; (2) the FC reorganization of CAL and CPL in some survived regions are significantly correlated with the sensorimotor/executive and high-order cognitive functions across stroke participants, respectively.

## Materials and methods

### Participants

The present study was conducted between February 1, 2018, and April 30, 2022. The Ethics Committee of Drum Tower Hospital, Nanjing University School of Medicine, approved this work. The study procedures were conducted in accordance with the Declaration of Helsinki, and written informed consent was obtained from all participants or their legal guardians as appropriate.

For the stroke participants, the inclusion criteria were as follows: (1) first-episode and subcortical stroke confirmed by CT or MRI; (2) age > 18 years; (3) right-handedness before stroke; (4) disease duration of stroke ≥ 3 months; the exclusion criteria were as follows: (1) contraindication for MRI; (2) suffering from neuropsychiatric disorders other than stroke, such as anxiety disorders, major depressive disorders, schizophrenia, and bipolar disorder; (3) unstable medical conditions, such as severe atrial fibrillation; (4) aphasia that hindered meaningful communication and assessment; (5) received transcranial electromagnetic and ultrasound stimulation; (6) addiction to tobacco, alcohol, or other drugs; and (7) incomplete information.

For the healthy participants, the inclusion criteria were as follows: (1) comparable age and level of education with stroke participants; (2) right-handedness; the exclusion criteria were as follows: (1) noticeable physical or neuropsychiatric disorders; (2) addiction to tobacco, alcohol, or other drugs; and (3) incomplete information.

### Behavioral instruments

Before MRI scanning, two behavioral assessments, including the Fugl-Meyer Assessment (Duncan et al., 1983), and the Chinese (Putonghua) version of the Oxford Cognitive Screen (OCS-P) (Hong et al., 2018), were evaluated by two therapists for each stroke participant separately. To assess the motor performance of stroke participants, the Fugl-Meyer Assessment was administered. Participants with non-acute stroke who achieve a score of 9 (sensitivity: 80.39%, specificity: 70%) to 10 (sensitivity: 97.62%, specificity: 89.66%) on the Fugl-Meyer Assessment Upper Extremity Scale (FMA-UE) are more likely to experience clinical improvement in disability (Arya et al., 2011). In addition, the Fugl-Meyer Assessment Lower Extremity Scale (FMA-LE) can differentiate the levels of lower extremity function in chronic stroke survivors with good sensitivity (0.87) and specificity (0.81) (Kwong and Ng, 2019). To assess the cognitive performance of stroke participants, the OCS-P subscales were administered. Our previous study revealed the OCS-P subscales, especially Picture Naming, Numerical Cognition, Praxis, and Delayed Recall and Recognition, have satisfactory content validity, substantive validity, construct validity, inter- and intrarater reliability, and known group discrimination for the stroke participants (Hong et al., 2018).

### Magnetic resonance imaging data acquisition

Images were acquired on a 3.0T MRI scanner (Philips Healthcare, Netherlands). Three-dimensional high-resolution T1-weighted images were acquired by the following parameters: repetition time = 9.9 ms, echo time = 4.6 ms, matrix = 256 × 256, slice thickness = 1 mm, field of view = 256 mm× 256 mm, 192 sagittal slices, voxel size = 1 mm× 1 mm× 1 mm, flip angle = 8°, and scan time = 6 min 47 s. T2-weighted images were collected using the following parameters: repetition time = 4,000 ms, echo time = 91 ms, matrix = 230 × 230, slice thickness = 5 mm, field of view = 230 mm× 230 mm, 30 axial slices, voxel size = 1 mm× 1 mm× 5 mm, flip angle = 90°, and scan time = 1 min 4 s. Resting-state fMRI data were acquired by the following parameters: repetition time = 2,000 ms, echo time = 30 ms, matrix = 64 × 64, slice thickness = 4 mm, field of view = 192 mm× 192 mm, voxel size = 3 mm× 3 mm× 4 mm, flip angle = 90°, 38 axial slices, 230 volumes, and scan time = 8 min 08 s.

### Lesion overlap analysis

**FIGURE 1:**
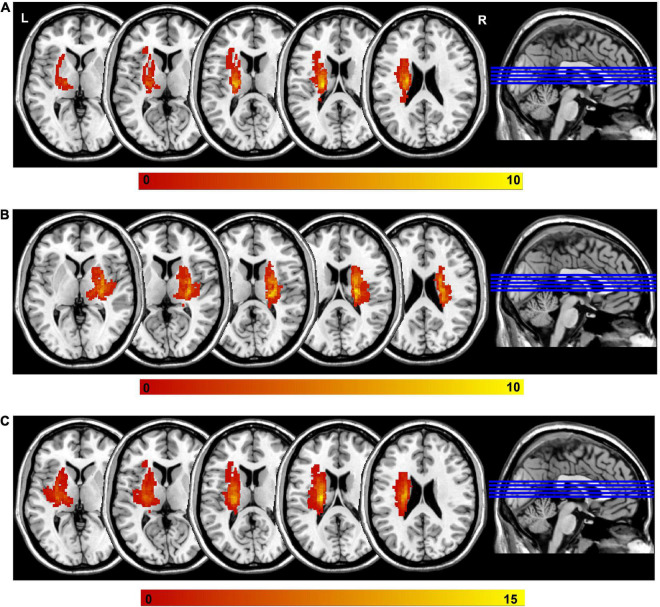
Lesion overlap across stroke participants. **(A)** The left-sided lesions; **(B)** the right sided lesions; **(C)** the overlapping of all lesions by mirroring the right lesions to the left side. Color bar indicates the subject number of lesion overlap. L, left; R, right.

The lesion volume of each participant was determined by two experienced neuroradiologists. One neuroradiologist manually outlined the profiles of the lesions on T2-weighted images slice by slice using the software MRIcron,^1^ and then the other one confirmed the lesions. In the process of identifying the lesion of the MRI data, both of them were blinded to the clinical data. The lesion overlap maps and locations for stroke participants were shown in Figure 1 and Supplementary Table 1, respectively.

### Image preprocessing

The preprocessing analyses were performed in combination with stringent motion artifact correction procedures using Statistical Parametric Mapping (SPM8)^2^ and Data Processing Assistant for Resting-State fMRI (DPARSF)^3^ (Yan et al., 2016). The image preprocessing procedure of the present study partly referred to our previous research (Zhao et al., 2018a,b; Hong et al., 2019). First, we discarded the first 10 volumes for each participant. The remaining 220 images were performed slice timing and head motion corrections. The criterium of head motion was defined as more than 2.5 mm of translation or greater than 2.5 degrees of rotation in any direction. Next, we regressed out the linear trend, mean white matter and cerebrospinal fluid signals, and the 24 head motion covariates (Friston et al., 1996) from each voxel’s time course. Subsequently, the functional images were normalized by using a diffeomorphic anatomical registration through exponentiated lie algebra (DARTEL) method and were resampled every 3 mm using the parameters estimated during unified segmentation. Then, the normalized images were spatially smoothed using an isotropic Gaussian filter at full width at a half maximum (FWHM) of 6 mm. Finally, we conducted the temporal bandpass filter (0.01–0.1 Hz).

### Functional connectivity analysis

**FIGURE 2:**
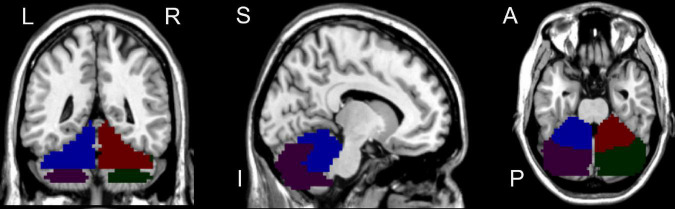
Seed regions of interest for functional connectivity. Blue region represents left cerebellum anterior lobe; Purple region represents left cerebellum posterior lobe; Red region represents right cerebellum anterior lobe; Green region represents right cerebellum posterior lobe. L, left; R, right; S, superior; I, inferior; A, anterior; P, posterior.

We selected the seed region of interest in the bilateral CAL and CPL (Figure 2) by xjView.^4^ Then, a voxel-wise FC analysis of each region of interest was performed on the resting-state data for all subjects. First, the blood-oxygen-level-dependent time series of the voxels within each seed region were averaged to obtain the reference time series for the seed region. Second, for each subject and each seed region, a correlation map was obtained by computing the correlation coefficients between the reference time series and the time series of the rest of the whole brain voxels. Finally, the correlation coefficients were converted to *z*-values using Fisher’s r-to-z transformation to improve the normality (Lowe et al., 1998).

### Statistical analysis

Statistical analysis of the demographic characteristics and clinical assessments was performed by using the Statistical Package for Social Sciences (SPSS) version 21 for Windows. To detect the alterations of FC in the CAL and CPL, a two-sample *t*-test with age, gender, education level, and mean frame-wise displacement as covariates were performed between stroke participants and HCs (GRF correction with a voxel-level *p* < 0.001 and a cluster-level *p* < 0.05). Moreover, to determine the relationship between the FC and clinical assessments, a partial correlation analysis was performed between the FC and FMA-UE, FMA-LE, and OCS-P subscales (Picture Naming, Numerical Cognition, Praxis, and Delayed Recall and Recognition) controlling for age, gender, education level, mean frame-wise displacement, and lesion volume across all stroke participants.

## Results

### Demographic characteristics and clinical assessments

A total of six eligible participants were excluded due to incomplete MRI scans for personal reasons (two post-stroke participants and two HCs) and excessive head motion (two post-stroke participants). Finally, thirty-six stroke participants with chronic subcortical stroke and thirty-eight HCs matched in gender, age, education level, mean frame-wise displacement, and handedness were included in the final analysis. The detailed demographic characteristics and clinical assessments of stroke participants and HCs were displayed in Table 1.

**TABLE 1:** Demographic characteristics and clinical assessments data of all participants in this study.

Baseline characteristics		Stroke participants (*n* = 36)	Healthy controls (*n* = 38)	*p-*value
Age (M ± SD, years)^a^		56.72 ± 9.59	58.53 ± 7.64	0.37
Gender (female: male, *n*)^b^		3: 33	4: 34	0.75
Education (M ± SD, years)^a^		9.89 ± 2.77	9.68 ± 3.10	0.77
Hand dominance (left: right, *n*)		0: 36	0: 38	–
Duration of illness (M ± SD, months)		14.22 ± 10.52	–	–
Mean frame-wise displacement (M ± SD)^a^		0.17 ± 0.16	0.14 ± 0.11	0.32
Lesion side (left: right, *n*)		17: 19	–	
Lesion volume (M ± SD, ml)		3.14 ± 2.98	–	–
FMA	FMA-UE (M ± SD, scores)	47.50 ± 18.55	–	–
	FMA-LE (M ± SD, scores)	27.64 ± 6.56	–	–
OCS-P	Picture Naming (M ± SD, scores)	3.71 ± 0.62	–	–
	Numerical Cognition (M ± SD, scores)	6.19 ± 1.41		–
	Praxis (M ± SD, scores)	10.89 ± 2.90	–	–
	Delayed Recall and Recognition (M ± SD, scores)	7.14 ± 1.40	–	–

^a^represents independent *t*-test.

^b^represents Chi-Square test. n, number; M, mean; SD, standard deviation; FMA, the Fugl-Meyer Assessment; FMA-UE, the Fugl-Meyer Assessment Upper Extremity Scale; FMA-LE, the Fugl-Meyer Assessment Lower Extremity Scale; OCS-P, the Chinese (Putonghua) version of the Oxford Cognitive Screen.

### Between-group difference in functional connectivity

**FIGURE 3:**
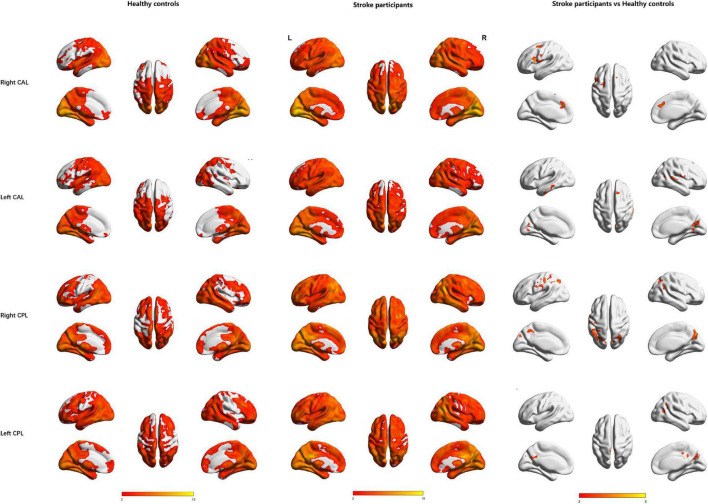
Regions showing significant functional connectivity with the bilateral cerebellar anterior lobe and cerebellar posterior lobe in the healthy controls (1st row) and stroke participants (2nd row) and regions showing significant differences between groups (3rd row). L, Left; R, Right; CAL, cerebellar anterior lobe; CPL, cerebellar posterior lobe.

The right CAL exhibited increased FC in the left prefrontal lobe (IFG, medial frontal gyrus (MedFG) and MFG) and PreCG, while the left CAL showed increased FC in the right MFG, right superior frontal gyrus, left inferior temporal gyrus, right superior temporal gyrus, and right lingual gyrus in the stroke participants compared to HCs (Table 2 and Figure 3).

**TABLE 2:** Regions showing significantly different functional connectivity (FC) in cerebellum anterior lobe (CAL) between the stroke participants and healthy controls.

Regions	MNI coordinates			Cluster size (Voxel number)	*t-*value
	*x*	*y*	*z*		
**Right cerebellum anterior lobe**					
**Stroke participants > healthy controls**					
Left precentral gyrus	−51	12	15	96	5.09
Left inferior frontal gyrus				92	
Left middle frontal gyrus	0	9	66	67	4.89
Left medial frontal gyrus	−6	36	27	100	4.98
**Stroke participants < healthy controls**					
None					
**Left cerebellum anterior lobe**					
**Stroke participants > healthy controls**					
Left inferior temporal gyrus	−54	−18	−33	143	4.82
Right lingual gyrus	12	−63	18	158	5.06
Right superior temporal gyrus	54	3	45	187	5.26
Right superior frontal gyrus				145	
Right middle frontal gyrus				112	
**Stroke participants < healthy controls**					
None					

MNI, Montreal Neurological Institute.

The right CPL displayed increased FC in the left PreCG, bilateral IPL, and right precuneus, while the left CPL exhibited increased FC in the bilateral middle cingulum gyrus, right angular gyrus, bilateral precuneus, and right MTG in the stroke participants compared to HCs (Table 3 and Figure 3).

**TABLE 3:** Regions showing significantly different functional connectivity (FC) in cerebellum posterior lobe (CPL) between the stroke participants and healthy controls.

Regions	MNI coordinates			Cluster size (Voxel number)	*t*-value
	*x*	*y*	*z*		
**Right cerebellum posterior lobe**					
**Stroke participants > healthy controls**					
Left inferior parietal lobule	−51	−48	42	260	4.93
Left precentral gyrus				102	
Right precuneus	36	−63	39	442	5.47
Right inferior parietal lobule				183	
**Stroke participants < healthy controls**					
None					
**Left cerebellum posterior lobe**					
**Stroke participants > healthy controls**					
Left precuneus	0	−36	27	85	4.88
Right precuneus				78	
Left middle cingulum gyrus				72	
Right middle cingulum gyrus				71	
Right angular gyrus	51	−63	18	91	4.78
**Stroke participants < healthy controls**					
None					

MNI, Montreal Neurological Institute.

Bilateral CAL and CPL exhibited no significantly decreased FC in the stroke participants compared to HCs (Tables 2, 3 and Figure 3).

### Correlations between functional connectivity and clinical assessment scores

Partial correlations revealed significant negative correlations between the FC and OCS-P subscale scores in the stroke participants. The FC between right CAL and left MedFG was negatively correlated with Praxis scores (*r* = −0.438, *p* = 0.015) and that between right CPL and right precuneus was negatively correlated with Delayed Recall and Recognition scores (*r* = −0.411, *p* = 0.024). However, these correlations did not survive after the FDR correction of *p* < 0.05.

## Discussion

In the present study, we explored the FC alternations of the CAL and CPL and their correlations with motor and cognitive functions in chronic subcortical stroke participants. The main findings of this study are: (1) relative to HCs, the CAL exhibited increased FCs in the prefrontal cortex, superior/inferior temporal gyrus, and lingual gyrus, while the CPL showed increased FCs in the IPL, precuneus, and cingulum gyrus; (2) the FCs between the right CAL and the left MedFG and between the right CPL and the right precuneus were negatively correlated with executive and memory functions across all post-stroke participants, respectively. Collectively, these findings provide new insight into the specific neurophysiological mechanisms of the functional reorganization of the cerebellar regions and their relationships with behavior performance underlying chronic subcortical stroke, which may facilitate precise modulation of targeted regions and thus enhance post-stroke dysfunction recovery.

### Functional connectivity alterations in the cerebellum anterior lobe

The CAL, grouped as cerebellar lobules I-V (Stoodley and Schmahmann, 2009), is often viewed as a unit for the performing sensorimotor function (Stoodley and Schmahmann, 2010; Sokolov et al., 2017), such as ongoing movements learning, control, and coordination (Diedrichsen et al., 2007; Deluca et al., 2014; Marko et al., 2015). Previous studies have focused on the functional plasticity of CAL in stroke participants (Wang et al., 2010; Yin et al., 2014). For instance, Yin et al. (2014) reported that chronic subcortical stroke participants showed significantly increased FC between the right anterior inferior cerebellum and left prefrontal cortex (MFG) in comparison to HCs. In the present study, we found increased FC between the CAL and the prefrontal cortex (e.g., IFG, MedFG, MFG, and superior frontal gyrus), superior/inferior temporal gyrus, and lingual gyrus in the post-stroke participants compared to HCs. These findings provide evidence that subcortical stroke lesions may activate the FC in CAL at the chronic stage. In addition to participating in sensorimotor function (Sokolov et al., 2017), the CAL is also considered as a complex role in cognitive function (Chao et al., 2021). In line with the previous evidence that performance on executive function was associated with damage to the CAL (Stoodley et al., 2016), the present study found that chronic subcortical stroke participants showed the FC between right CAL and left MedFG negatively correlated with executive function assessed by the Praxis in the OCS-P tool (Hong et al., 2018), although did not survive after FDR correction. In summary, the present findings might jointly demonstrate an inhibitory role of hyper-reorganization in CAL in motor-related high-order cognitive function, specifically executive function, among chronic subcortical stroke participants.

### Functional connectivity alterations in the cerebellum posterior lobe

The CPL, which comprises cerebellar lobules VI-IX (Schmahmann et al., 2000), has been reported to principally engage in the complex cognitive process (Schmahmann and Caplan, 2006; Reetz et al., 2012), such as the regulation of language and working memory (Nitschke et al., 2005; Schmahmann et al., 2019). Previous studies have revealed the functional alternations of the CPL in stroke (Li et al., 2013; Tang et al., 2016; Fan et al., 2019; Zhang et al., 2022). For example, Li et al. (2013) found, relative to HCs, stroke participants showed reduced FCs between the bilateral cerebellar lobule IX and the ipsilesional prefrontal cortex (medial part of the prefrontal cortex and dorsolateral prefrontal cortex) and lateral parietal cortex. Tang et al. (2016) reported decreased FCs between the right CPL and left IFG, PreCG, IPL, and MTG in stroke participants compared with HCs. These findings demonstrated that the functional reorganization in the CPL mainly concentrated in the frontoparietal region and partial temporal cortex in stroke participants. Similarly, the present study found increased FCs between CPL and PreCG, IPL, angular gyrus, MTG, precuneus as well as cingulum gyrus in post-stroke participants compared with HCs. Furthermore, we found the bilateral CPL both exhibited increased FCs patterns, which is in line with the results from Yin et al. (2012) that chronic stroke participants showed increased FC between the ipsilesional CPL and the contralesional primary motor cortex compared with HCs. Moreover, previous studies indicated that the CPL was involved in cognitive impairments (Schmahmann, 2004), such as visuospatial working memory (Nitschke et al., 2005). Similarly, this present study found that the FC between the right CPL and right precuneus was negatively related to Delayed Recall and Recognition in chronic subcortical stroke participants, although it did not survive after FDR correction. Hence, we hypothesized that the increased FC in the bilateral CPL with frontoparietal and temporal and the negative correlations between FC in CPL and memory functions collectively revealed that hyper-reorganization in the CPL might be involved in behavior impairments in chronic subcortical stroke participants.

### Limitations

Several limitations in the present study should be noted. First, our study was cross-sectional; therefore, we were unable to capture dynamic FC alterations and their relationships with behavior performance in chronic subcortical stroke participants. Future longitudinal studies are needed to investigate these dynamic abnormalities. Second, the sample size was relatively small, and there was a heavy male predominance in the present study. Although we controlled for gender as a nuisance covariate in the statistical analysis, a study with a larger sample size and the appropriate gender ratio would be necessary to confirm our findings in the future. Third, the slice thicknesses are large for the acquisition of resting-state fMRI and T2-weighted data. Using the developed multiband/multiplexed echo planar imaging methods (Feinberg et al., 2010; Moeller et al., 2010) may enhance the quality of fMRI data with unprecedented sampling rates for full-brain coverage. Moreover, we will acquire T2-weighted images with isotropic 1 mm in future studies to map the lesion accurately. Finally, the CAL and CPL can actually be divided into lobules I-V and VI-X, respectively, but the current seed region of interest is selected in the CAL and CPL by averaging the time courses within the brain region, and this may overlook the functional contribution of each subregion in the cerebellum. In the future, we may consider concentrating on the changes in FC of subregions in the cerebellum in stroke participants.

## Conclusion

In the present study, we explored FC alternation in the cerebellar subregions in chronic stroke participants with subcortical lesions. We found the CAL and CPL showed different FC increases with cerebral regions in chronic stroke participants relative to HCs, and the FCs in the right CAL and CPL were negatively correlated with executive and memory functions, respectively. Taken together, our findings provide complementary evidence to further understand the neurophysiological mechanisms underlying behavioral performance after stroke, which could motivate the application of noninvasive brain stimulation (e.g., transcranial magnetic stimulation) in chronic stroke participants.

## Data availability statement

The raw data supporting the conclusions of this article will be made available by the authors, without undue reservation.

## Ethics statement

The studies involving human participants were reviewed and approved by the Ethics Committee of Drum Tower Hospital, Nanjing University School of Medicine. The patients/participants provided their written informed consent to participate in this study.

## Author contributions

GX, WH, and FS designed the study. WH, XZ, ZL, ML, ZY, YW, and BY collected the data. WH and FS contributed to the data analysis. WH, YD, and FS drafted the manuscript. WH, FS, RX, GX, and MW revised the manuscript. All authors contributed and approved the final manuscript.

## Footnotes

1
https://www.nitrc.org/projects/mricron

2
http://www.fil.ion.ucl.ac.uk/spm

3
http://rfmri.org/DPARSF

4
https://www.alivelearn.net/xjview

## Abbreviations

CAL: cerebellum anterior lobe
CPL: cerebellum posterior lobe
FC: functional connectivity
FMA-LE: Fugl-Meyer Assessment Lower Extremity Scale
FMA-UE: Fugl-Meyer Assessment Upper Extremity Scale
HCs: healthy controls
IFG: inferior frontal gyrus
IPL: inferior parietal lobule
MedFG: medial frontal gyrus
MFG: middle frontal gyrus
MTG: middle temporal gyrus
OCS-P: Chinese (Putonghua) version of the Oxford Cognitive Screen
PreCG: precentral gyrus.
