# Supplementary material for: Altered cerebellar functional connectivity in chronic subcortical stroke patients
Source: Front Hum Neurosci. 2022 Nov 11;16:1046378. doi: 10.3389/fnhum.2022.1046378 (PMC9691772; doi:10.3389/fnhum.2022.1046378)
Supplement: Supplementary file 1 [file Table_1.DOCX]

**Table S1. The lesion locations of all post-stroke participants.**

| **Post-stroke Participants** | **Lesion side (Left/Right)** | **Lesion locations** |
| --- | --- | --- |
| Subject 01 | Left | Corona radiata |
| Subject 02 | Left | Corona radiata, Internal capsule, Thalamus |
| Subject 03 | Right | Corona radiata, Caudate nucleus, Internal capsule |
| Subject 04 | Left | Corona radiata, Internal capsule |
| Subject 05 | Left | Internal capsule, Thalamus |
| Subject 06 | Right | Corona radiata |
| Subject 07 | Right | Internal capsule, Thalamus |
| Subject 08 | Right | Thalamus |
| Subject 09 | Right | Internal capsule, Thalamus |
| Subject 10 | Left | Corona radiata |
| Subject 11 | Left | Corona radiata, Caudate nucleus, Putamen, Internal capsule |
| Subject 12 | Left | Corona radiata, Internal capsule, Thalamus |
| Subject 13 | Left | Corona radiata, Internal capsule, Thalamus |
| Subject 14 | Left | Thalamus |
| Subject 15 | Left | Corona radiata, Internal capsule, Thalamus |
| Subject 16 | Right | Internal capsule, Putamen |
| Subject 17 | Right | Internal capsule, Globus pallidus, Putamen, Thalamus |
| Subject 18 | Right | Internal capsule, Globus pallidus |
| Subject 19 | Left | Corona radiata, Internal capsule, Thalamus |
| Subject 20 | Left | Corona radiata, Internal capsule, Thalamus |
| Subject 21 | Left | Corona radiata, Internal capsule |
| Subject 22 | Right | Corona radiata, Internal capsule, Putamen, Thalamus |
| Subject 23 | Left | Corona radiata, Internal capsule, Thalamus |
| Subject 24 | Right | Corona radiata, Internal capsule, Globus pallidus, Thalamus |
| Subject 25 | Right | Putamen, Internal capsule |
| Subject 26 | Left | Corona radiata, Internal capsule, Thalamus |
| Subject 27 | Left | Thalamus |
| Subject 28 | Right | Corona radiata |
| Subject 29 | Right | Corona radiata |
| Subject 30 | Right | Corona radiata, Globus pallidus, Putamen, Internal capsule |
| Subject 31 | Right | Internal capsule, Globus pallidus, Putamen |
| Subject 32 | Right | Corona radiata, Internal capsule, Thalamus |
| Subject 33 | Right | Corona radiata, Internal capsule |
| Subject 34 | Right | Corona radiata, Internal capsule, Putamen, Thalamus |
| Subject 35 | Right | Corona radiata, Globus pallidus |
| Subject 36 | Right | Corona radiata, Globus pallidus |
